# On the Importance of Attention and Augmentations for Hypothesis Transfer in Domain Adaptation and Generalization

**DOI:** 10.3390/s23208409

**Published:** 2023-10-12

**Authors:** Rajat Sahay, Georgi Thomas, Chowdhury Sadman Jahan, Mihir Manjrekar, Dan Popp, Andreas Savakis

**Affiliations:** Rochester Institute of Technology, Rochester, NY 14623, USA; rs6287@g.rit.edu (R.S.); sj4654@g.rit.edu (C.S.J.);

**Keywords:** domain adaptation, domain generalization, vision transformers, convolutional neural networks

## Abstract

Unsupervised domain adaptation (UDA) aims to mitigate the performance drop due to the distribution shift between the training and testing datasets. UDA methods have achieved performance gains for models trained on a source domain with labeled data to a target domain with only unlabeled data. The standard feature extraction method in domain adaptation has been convolutional neural networks (CNNs). Recently, attention-based transformer models have emerged as effective alternatives for computer vision tasks. In this paper, we benchmark three attention-based architectures, specifically vision transformer (ViT), shifted window transformer (SWIN), and dual attention vision transformer (DAViT), against convolutional architectures ResNet, HRNet and attention-based ConvNext, to assess the performance of different backbones for domain generalization and adaptation. We incorporate these backbone architectures as feature extractors in the source hypothesis transfer (SHOT) framework for UDA. SHOT leverages the knowledge learned in the source domain to align the image features of unlabeled target data in the absence of source domain data, using self-supervised deep feature clustering and self-training. We analyze the generalization and adaptation performance of these models on standard UDA datasets and aerial UDA datasets. In addition, we modernize the training procedure commonly seen in UDA tasks by adding image augmentation techniques to help models generate richer features. Our results show that ConvNext and SWIN offer the best performance, indicating that the attention mechanism is very beneficial for domain generalization and adaptation with both transformer and convolutional architectures. Our ablation study shows that our modernized training recipe, within the SHOT framework, significantly boosts performance on aerial datasets.

## 1. Introduction

In recent years, the emergence of attention-based transformer models [1,2,3] has stimulated interest in new architectures that have achieved state-of-the-art results on a wide variety of computer vision tasks [4,5,6]. Along with these exciting developments and the growing interest in deploying models in practice, there is a need to investigate the robustness of attention-based models when deployed to new settings. This is especially useful when supervised transfer learning is not possible due to a lack of labels in the new domain. In this paper, we benchmark the generalization and adaptation performance of transformer models for vision and compare them with convolutional neural networks (CNNs) under distribution shifts between the training and testing data. Our in-depth analysis compares three members of the transformer family, vision transformer (ViT) [1], shifted window transformer (SWIN) [2] and dual attention vision transformer (DAViT) [3], against convolution-based architectures ResNet50 [7], HRNet [8], and the more recently introduced attention-based ConvNeXt [9]. While convolutions excel at capturing local patterns in the input domain (e.g., edges and contours) [10], self-attention mechanisms have been shown to effectively learn global patterns, such as the relations between distant parts of an image [1]. We explore the performance of these models through the lens of the source hypothesis transfer (SHOT) framework.

In the domain generalization and adaptation setting, the goal is (a) to learn robust feature representations for the source distribution that generalize well to the target distributions and (b) to adapt to the unlabeled target domain. In this paper, we consider domain adaptation benchmarks on two distinct visual perspectives or views: (i) images of objects at ground level found in standard datasets and (ii) aerial imagery from drones or satellites. Although previous works have looked at the performance of different domain transfer techniques on generic and aerial datasets [11,12], our work explores the merits of transformer vs. convolutional backbone architectures.

Our results on standard datasets, such as Office-Home [13] and DomainNet [14], serve as a benchmark for future work, while adaptation on aerial imagery deals with its own unique set of challenges. With changes in rotation, scale, illumination, and noise due to different sensor and viewpoint characteristics, dedicated models need to be trained on aerial data. The situation is further exacerbated by challenging conditions, including lower inter-class variance, weather-related disturbances, and greater variations in the orientation of objects with respect to the background. Our work examines how different convolutional and self-attention based models perform when presented with such challenging tasks.

In this paper, we present a broad comparison of the performance of different types of architectures for domain generalization and unsupervised domain adaptation tasks. Our systematic experiments on domain adaptation with the SHOT framework led to the following observations and contributions:

- Self-attention based transformer models ViT, DAViT and SWIN generally outperform standard convolutional models ResNet and HRNet on both generalization and adaptation tasks.
- The newer attention-based convolutional network ConvNext is on par or better than transformer models (ViT, SWIN, and DAViT) and does better than standard convolutional models (ResNet and HRNet).
- Using the SWIN architecture as a backbone results in better performance compared to ViT and DAViT, especially for the adaptation task.
- Image augmentations, such as RandAugment and RandomErasing, used for adaptation may result in lower performance for standard datasets but improve the performance of models on aerial datasets.

The remainder of this paper is organized as follows. Section 2 focuses on a brief literature review of prior works done in this area. Section 3 describes the different architectures as well as the SHOT framework that we employ to carry out the adaptation process. Section 4 discusses our experimental setup, which includes the different types of datasets that we used and our *modern training recipe* of data augmentations. Section 5 provides comprehensive results from the study, while additional results are presented in the appendix. Finally, Section 6 presents final remarks and conclusions based on our evaluation.

## 2. Related Work

### 2.1. Domain Transfer

Distribution shift or domain shift occurs when the training (source) distribution differs from that of the test (target) distribution, leading to a significant degradation in the performance of deployed source-trained systems. A transductive transfer learning technique [15], termed domain transfer, aims at mitigating the domain shift by leveraging knowledge from the labeled source domain to learn effective features for the unlabeled target domain. A multitude of domain alignment methods have been proposed in recent years. Earlier approaches exploit the shift-invariant information of feature embeddings to match different target domains to the source domain [16,17,18]. Other methods employ adversarial methods to align source and target features [19,20], or minimize the discrepancy across different domain distributions in the feature space [21,22]. Others make use of entropy optimization methods [23,24] or minimize the discrepancy between joint features by means of optimal transport [25].

### 2.2. Source-Free Domain Adaptation

A different paradigm of domain adaptation methods called source-free domain adaptation (SFDA) [26,27,28,29] has emerged in recent years driven by privacy concerns [30] in traditional DA techniques. In SFDA, practitioners only have access to the target data and a source data trained model during adaptation. A prominent SFDA method is source-free hypothesis transfer (SHOT) [31], which utilizes information maximization and entropy minimization via a self-supervised pseudo-labeling strategy to adapt the source-trained features to the target domain features. Other methods [26,29] use generative networks to model the distribution of target data by generating target-style images to skew the source distribution, which enhances the model performance on the target domain. G-SFDA [32] exploits the neighborhood structure of data by activating different channels in the network for different domains. $A^{2}$-Net [28] uses an adversarial training strategy to align the two domains, while SoFA [27] employs a variational autoencoder to encode the target distribution in latent space, and the generation process from the predicted classes to input data is modeled to infer latent features for alignment.

### 2.3. Vision Transformers for Domain Adaptation

Transformers [33] were first proposed for natural language processing (NLP) and soon demonstrated impressive performance on various tasks such as text classification and machine translation [34,35]. Much of the success of transformer models is due to their ability to capture long-range dependencies through the self-attention mechanism. Spurred by the success of transformers in NLP, vision transformers (ViT) [1] were introduced for image-centric tasks and showed great promise. In a traditional ViT model, the processing stages are applied to a sequence of fixed, non-overlapping image patches, and a self-attention mechanism is used to encode context. ViT and its variants have demonstrated wide applicability in object detection [4,36], segmentation [5,37], and video understanding [6]. The success of vision transformers is attributed to global context modeling [33] by pretraining on large-scale data, as opposed to relying on image-specific inductive biases, e.g., translation equivariance employed by traditional CNNs.

However, despite their success on different vision tasks, there has been little work on exploring the domain transferability of vision transformer backbones for UDA tasks compared to CNNs. To this end, we benchmark vision transformer backbones against CNN backbones using the SHOT framework under a uniform set of input parameters, enabling a direct comparison between the two families of feature extraction architectures for domain generalization and adaptation for image classification.

We note that there are several contemporary works [38,39,40] that apply different self-attention backbones, e.g., DeiT [41] and SWIN [2], for the purposes of UDA. Specifically, ref. [38] uses a weight-sharing triple-branch transformer to utilize the benefits of self- and cross-attention mechanisms for domain alignment. Ref. [40] focuses on both transferable and discriminative features of different domains by injecting learned transferability into the attention blocks. Different from these, our work explores the efficacy of off-the-shelf vision transformer feature extractors and their global self-attention mechanism on unsupervised DA. We also compare their performance with that of CNN models utilizing hierarchical receptive fields.

## 3. Methodology

In this section, we take a deeper look at the (SHOT) [31] framework that we employ for source-free unsupervised domain adaptation (UDA), as well as the different CNN and transformer backbones we utilize for feature extraction.

### 3.1. Source Hypothesis Transfer (SHOT)

**Figure 1:**
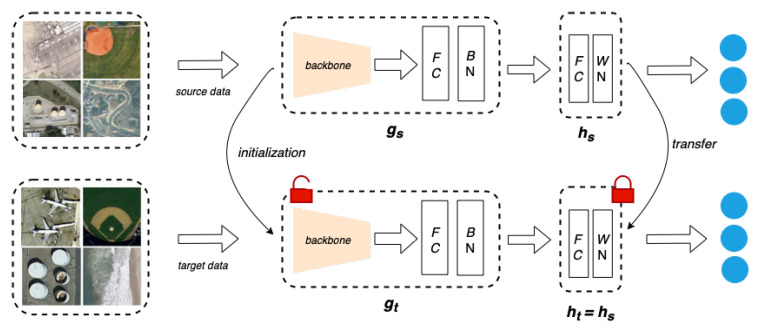
An overview of the source hypothesis transfer (SHOT) architecture [31].

The SHOT framework proposes a discrepancy-based domain adaptation technique by employing hypothesis transfer learning [42] from a source domain $D_{s}$ to a target domain $D_{t}$. An overview of the overall architecture can be seen in Figure 1. For a vanilla UDA task, we are provided with $n_{s}$ labeled samples $\left\{\left(x_{s},y_{s}\right)\in \left(X_{s},Y_{s}\right)\right\}$ from the source domain and $n_{t}$ samples $\left\{x_{t}\in X_{t}\right\}$ from the target domain. The goal is to learn a mapping function $f_{t}:X_{t}\to Y_{t}$ to determine the corresponding labels $\left\{y_{t}\in Y_{t}\right\}$ for the target domain.

SHOT initially trains the source feature extractor $\left(g_{s}\right)$ and the source hypothesis $\left(h_{s}\right)$ on the source data $D_{s}$ to learn the feature mapping $\left(f_{s}:X_{s}\to Y_{s}\right)$. The feature extractor $g_{s}:X_{s}\to R^{d}$ encodes input images into *d*-dimensional feature embeddings, and the hypothesis module $h_{s}:R^{d}\to R^{k}$ takes the embeddings and returns *k*-dimensional logits, where *k* is the total number of classes in the dataset. SHOT utilizes categorical cross-entropy loss with label smoothing [43] for the source-training procedure. Label smoothing helps to create soft class boundaries in the feature space. The overall source training loss can be mathematically defined as

(1) $$L_{src}\left(f_{s};X_{s},Y_{s}\right)=-E_{\left(x_{s},y_{s}\right)\in \left\{X_{s},Y_{s}\right\}}\sum_{k=1}^{K}q_{k}\;log\;\delta_{k}\left(f_{s}\left(x_{s}\right)\right)$$

where $\delta_{k}\left(a\right)=\frac{exp(a_{k})}{\sum_{i}exp\left(a_{i}\right)}$ denotes the *k*-th element in the softmax output of a *K*-dimnsional vector *a*, and $q_{k}$ is the one-of-*k* encoding of $Y_{s}$.

During adaptation, the target feature extractor $\left(g_{t}\right)$ is initialized with the source-trained backbone and remains trainable during adaptation. The source hypothesis (classifier) $\left(h_{s}\right)$ is transferred as the target hypothesis $\left(h_{t}\right)$ and is kept frozen during the network adaptation. In the absence of source data during the adaptation process, SHOT utilizes information maximization (IM) loss to make the target outputs individually certain and globally diverse. In practice, IM loss is the combination of the following $L_{ent}$ and $L_{div}$ loss functions:

(2) $$\begin{array}{cc} L_{ent}\left(f_{t};X_{t}\right) & =E_{x_{t}\in X_{t}}\sum_{k=1}^{K}\;\delta_{k}\left(f_{t}\left(x_{t}\right)\right)log\;\delta_{k}\left(f_{t}\left(x_{t}\right)\right),\\ L_{div}\left(f_{t};X_{t}\right) & =\sum_{k=1}^{K}{\hat{p}}_{k}log\;{\hat{p}}_{k} \end{array}$$

where $f_{t}\left(x\right)=h_{t}\left(g_{t}\left(x\right)\right)$ is the *k*-dimensional output of each target sample; and $\hat{p}=-E_{x_{t}\in X_{t}}\left[\delta \left(f_{t}^{\left(k\right)}\left(x_{t}\right)\right)\right]$ is the mean output embedding of the whole target domain. SHOT further proposes a self-supervised, pseudo-labeling approach based on the cosine distances from each of the centroids in the target feature space. The overall objective function can be defined as

(3) $$\begin{array}{c} L_{adapt}\left(g_{t}\right)=L_{ent}\left(h_{t}\circ g_{t};X_{t}\right)+L_{div}\left(h_{t}\circ g_{t};X_{t}\right)-\\ \beta E_{\left(x_{t},{\hat{y}}_{t}\right)\in \left\{X_{t},{\hat{Y}}_{t}\right\}}\sum_{k=1}^{K}1_{\left[k={\hat{y}}_{t}\right]}\;log\;\delta_{k}\left(h_{t}\left(g_{t}\left(x_{t}\right)\right)\right) \end{array}$$

where ${\hat{y}}_{t}\in {\hat{Y}}_{t}$ are the pseudo-labels and β>0 is a hyperparameter.

Thus, the overall objective function ($L_{adapt}$ in Equation (3)) for the target feature extractor $\left(g_{t}\right)$ during adaptation can be defined as a weighted combination of the IM loss (defined in Equation (2)) and the self-supervised loss for the pseudo-labeling approach. β is the weighting hyperparameter, which defines the amount of influence each component would have on the overall function. $E_{x_{t}\in X_{t}}\left[\delta \left(f_{t}\left(x_{t}\right)\right)\right]$ is the mean output embedding of the entire target domain, where $f_{t}\left(x\right)=h_{t}\left(g_{t}\left(x\right)\right)$, and ${\hat{y}}_{t}$ are the pseudo-labels for the target domain generated by comparing the cosine distances from each centroid in the target embedding space.

### 3.2. Backbone Architectures

Since the transferability of the model is closely correlated with the performance of the specific architecture on downstream tasks [44], having a strong source-trained model is vital in order to maximize the adaptation accuracy. Given that our objective is to compare and contrast the performance of convolutional and self-attention-based architectures, we choose three models from each family summarized in Table 1. For convolutional neural networks (CNNs), we use ResNet [7], HRNet [8], and ConvNeXt [9]. The ConvNeXt architecture improves upon traditional convolutional architectures by employing multiple macro and micro-strategies. Inspired by architectures like SWIN [2] and ResNeXt [45], these techniques include adding grouped and depthwise convolutions, varying the kernel size and number of convolutional blocks at each stage, and adding a separate 2×2 convolutional layer for spatial downsampling. Another major change in ConvNeXt is increasing the filter size from 3×3 to 7×7 to allow each convolutional layer to have a more global context. The authors also substituted batch normalization with layer normalization [46] (as seen in transformers), which further increases accuracy on image classification tasks.

For our study on self-attention based models, we selected three models based on their competitive performance in computer vision tasks: vision transformers [1], SWIN transformers [2], and dual-attention vision transformers [3]. SWIN transformers use a hierarchical transformer with a sliding window strategy, where self-attention is computed within a local window. On the other hand, DAViT incorporates both spatial window attention and channel group attention, allowing it to capture both abstract features and global interactions between spatial positions within an image.

## 4. Experimental Setup

### 4.1. Benchmarking Datasets

In order to benchmark the performance of models over multiple scenarios, we conduct our experiments using two different categories of datasets. The first category includes Office-Home [13] and DomainNet [14], which are datasets with standard, ground-level views of commonly found objects across multiple domains. The second category of datasets comprises aerial imagery collected from satellites. Aerial datasets present unique challenges for cross-domain adaptation due to the different image characteristics based on ground sampling distances (GSDs), unique sensors used for data collection, and lower inter-class variation. The aerial datasets were introduced in [12] and utilize the shared classes of publicly available aerial datasets for classification. We provide more information on the individual datasets in Section 4.3.

### 4.2. Standard DA Datasets

For our experiments, we considered two standard DA datasets that portray objects at the ground level: Office-Home [13] and DomainNet [14]. We describe the characteristics of each of the datasets in more detail in the following subsections.

#### 4.2.1. Office-Home

**Figure 2:**
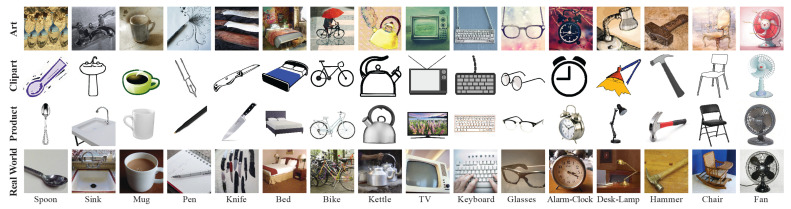
Sample images from Office-Home dataset. The figure displays examples from 16 of the 65 categories.

Office-Home [13] is a medium-sized dataset with 15,500 images consisting of 65 different image classes across four domains: art (Ar), clipart (Cl), product (Pr), and real world (Rw). Sixteen of the classes across four domains are shown in Figure 2.

#### 4.2.2. DomainNet

**Figure 3:**
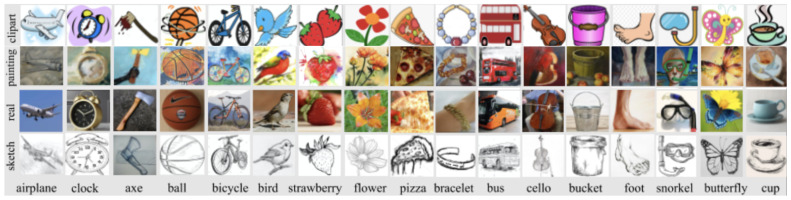
Sample images from the DomainNet-126 dataset. The figure displays examples from 10 classes across the four domains.

The DomainNet dataset [14] contains images of common objects in six different domains. All domains include 345 classes of everyday objects, such as bracelets, birds, and cellos. The domains include clipart (C), real world (R), sketch (S), painting (P), infograph (I), which are infographic images of a specific object, and quickdraw (Q), which are drawings from worldwide players of the game “Quick Draw!”. For the purposes of this study, we use DomainNet-126, which is a subset of the original DomainNet dataset with 126 classes across four domains: clipart, real world, sketch, and painting. We can see a sample of the dataset in Figure 3.

### 4.3. Aerial Datasets

We consider two aerial datasets for our domain adaptation problem. Since there is a lack of datasets that are specifically suited for our task, we follow a process similar to [12] in order to create our own datasets. To do so, we take four publicly available datasets and divide them into two pairs. We then utilize the shared classes between the datasets in each pair in order to represent the same object in a different domain.

#### 4.3.1. DOTA-xView

**Figure 4:**
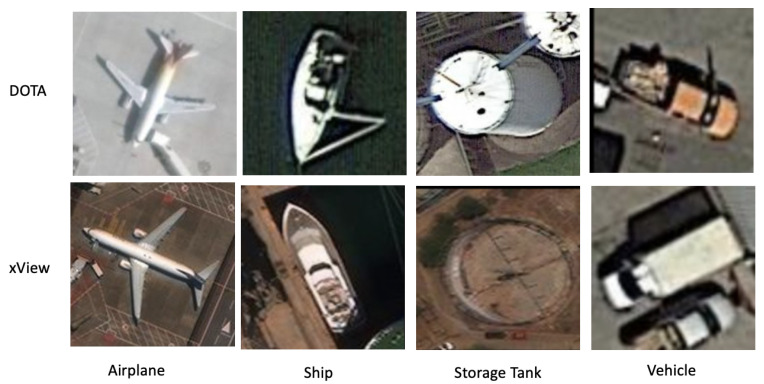
Sample images from our DOTA-xView dataset. The figure displays the 4 common classes across both the domains.

Our first aerial dataset for the domain adaptation task was formed by taking the classes common between the dataset for object detection in aerial images (DOTA) [47] and xView [48] datasets. A sample of our combined dataset can be seen in Figure 4. We take five shared classes and a varying number of samples from each class for our DOTA-xView DA dataset. More information can be found in Table 2.

The DOTA dataset [47] is a benchmark dataset created for performing object detection in aerial images. The images are mainly collated using data from Google Earth and the China Center for Resources—Satellite Data and Application. There are a total of 2086 high-resolution images ranging from 800 ×800 to 6000 × 6000 pixels. The dataset includes objects from 15 classes, and multiple objects of different classes may be present in the same image. This makes it difficult to perform classification accurately.

To overcome this limitation, each image is cropped around the bounding boxes to ensure it only has a single object corresponding to a specific class. The size of the cropped images ranges from 10 × 10 to 904 × 904. To further achieve optimal results, images smaller than 30 × 30 are discarded, and the number of images per class is restricted to 5000. We perform data augmentation for classes that do not meet this number by flipping the final images both horizontally and vertically.

For our experiments, we select the following five classes: large vehicle, plane, ship, small vehicle, and storage tank. We combine the large and small vehicle classes to form the vehicle class. We randomly delete half the images to keep the total number of images per class constant, which prevents overfitting.

The xView dataset was created as part of the 2018 xView Detection Challenge [48]. It contains approximately 1 million object samples divided across 60 classes. The images in this dataset were captured using the WorldView-3 satellite and have a resolution of 0.3 m/pixel. The objects within each image in this dataset vary in size from 3 m to greater than 3000 m.

Similar to DOTA, xView has multiple instances of objects belonging to different classes in each high-resolution image. Therefore, we apply similar pre-processing steps by cropping each image around the bounding boxes and discard any cropped images less than 30 × 30 in size. We also restrict the number of images per class to 5000 and perform data augmentation by horizontally and vertically flipping images for classes that do not meet this number.

The final xView partition consists of the same classes as the DOTA dataset described above: large vehicle, plane, ship, small vehicle, and storage tank. As with DOTA, we combine and randomly delete half the images of large and small vehicles to form a singular vehicle class.

#### 4.3.2. AID-UCM

**Figure 5:**
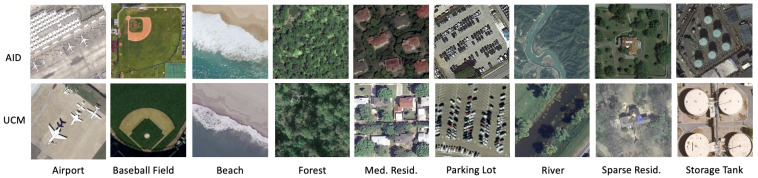
Sample images from the AID-UCM dataset. The figure displays the nine common classes across both domains.

Our second aerial dataset for the domain adaptation is formed by taking the classes common between the AID [49] and UCM [50] datasets, each of which we define in more detail below. We take nine shared classes and a varying number of samples from each class for our AID-UCM dataset. More information can be found in Table 3, and a sample of our final dataset can be seen in Figure 5.

The Aerial Image Dataset (AID) [49] was developed for the task of aerial scene classification by taking images from Google Earth. It contains a total of 10,000 aerial images divided across 30 classes. Each image measures 600 × 600 pixels and is annotated by experts in remote sensing image interpretation. The data consist of images taken in diverse geographic locations with variances in both time and season. The images in the dataset are obtained at multiple GSDs ranging from 8 m to 0.5 m. We select nine classes from AID for the purposes of our experiments: airport, parking, storage tank, beach, forest, river, baseball field, medium residential, and sparse residential.

The UC Merced Land Dataset (UCM) [50] is a publicly available image dataset of overhead land images meant for research purposes. It consists of 21 classes and has 100 images per class, measuring 256 × 256 pixels and having a resolution of 0.3 m/pixel in the RGB space. The images are downloaded from the United States Geological Survey (USGS) National Map from different urban US regions. The images selected contain a wide variety of spatial patterns, textures, and colors, making them ideal for scene classification.

Our partition of the UCM dataset consists of the same classes as the AID dataset described above: airport, parking lot, storage tank, beach, forest, river, baseball diamond, medium residential, and sparse residential.

### 4.4. Modernizing the Training Recipe with Data Augmentations

Self-attention models, in general, require a large amount of data for effective training [1]. The lack of sufficient data was addressed by employing a training recipe via a new image augmentation protocol. We take this protocol and formulate our new training recipe involving augmentations to the source domain data during training to improve generalization. We implement two data augmentation procedures:

- **RandAugment**: An automated data augmentation technique that uniformly samples operations from a set of augmentations, such as rotation, equalization, color jittering, solarization, translation, shearing, and altering physical characteristics, such as contrast, brightness, and sharpness.
- **Random erasing**: A rectangular region in the image is replaced with random values. Multiple levels of occlusions are created to help the models generate more robust features.

These two data augmentation techniques allow the models to extract a richer set of features from the input images and provide larger variations in the appearance of aerial images by artificially varying physical characteristics, such as object positioning and frame contrast. Inspired by the augmentations in ConvNeXt [9], our initial experiments considered two more augmentation techniques, Cutmix and Mixup, in addition to RandAugment and random erasing. Cutmix is based on interchanging patches between different images in the training set and Mixup is based on generating weighted combinations of random image pairs. However, our experiments in Table 4 show that combining RandAugment and random erasing with any additional augmentations does not improve performance but rather results in a significant degradation in accuracy for both convolutional and self-attention based models. Thus, going forward, we only use RandAugment and random erasing as our image augmentation techniques for our study.

In keeping with the new augmentation process, we modify the parameters of our framework slightly and run adaptation for 100 epochs during source training. We also tune the learning rate to 0.001 in order to prevent overfitting. To further validate the effectiveness of our modern training recipe, we compare our results with and without the use of these augmentations to determine if they help transformer models to generalize better.

Moreover, we find that our models benefit from a higher learning rate in the initial epochs and a lower learning rate during later epochs. Based on this observation, we use a linear learning rate decay during our training process. This allows us to achieve better convergence while training our models.

### 4.5. Setup of Architectures

In order to maintain consistency across the different architectures, we use an image size of 224×224 pixels across all of the backbones. We also use the base models of the SWIN, ViT, ConvNeXt, and DAViT architectures. All models used are pre-trained on ImageNet-1k. For the generalization task without the modernized training recipe, all models are trained for 20 epochs with a learning rate of 0.01. During adaptation, we maintain the same learning rate of 0.01 for standard datasets and use 0.001 for the aerial datasets. This is done in order to compensate for the increased ambiguity present in images in the aerial domain, which make the task of image classification considerably harder.

To analyze the effect of image augmentation on domain generalization, we compare two training recipes. Image augmentations are only applied on the source domain data when training models for generalization, and after training, the models adapt to the target data without augmentations. This is done because of the lack of labels in the target domain in order to prevent the models from extracting incorrect labels for the images.

## 5. Results and Discussion

In this section, we present the results of our experiments and compare the performance of different backbone architectures for unsupervised domain adaptation. We evaluate our model with and without the modern training recipe in Section 4.4 on both generic and aerial datasets.

The primary metric that we use to compare the models and architectures considered is classification accuracy, i.e., the ratio of the number of samples correctly classified by the model and the total number of predictions performed by the model. In our results, we report the ’mean percent accuracy’, which is the classification accuracy for each target domain averaged over all source–target pairs present in a particular dataset. We provide a more detailed presentation of our results over individual domains in Appendix A.

### 5.1. Results for Standard Datasets

The standard DA datasets in our experiments are Office-Home [13] and DomainNet [14]. The results in Table 5 show that the ConvNeXt backbone generalizes better than all the other models, closely followed by SWIN. For adaptation, ConvNeXt outperforms other models for Office-Home while coming just behind SWIN on DomainNet. More broadly put, self-attention based models, like ViT and SWIN, generalize and adapt better to other domains compared to convolutional backbones, like ResNet and HRNet. However, ConvNeXt breaks this trend by outperforming all other models under comparison, both convolutional and transformer-based. ConvNext [9] utilizes a combination of cardinality and depthwise convolutions, along with a separate 2×2 layer for spatial downsampling. The ConvNext architecture allows it to capture richer spatial information and learn more powerful representations that help it achieve better performance.

Table 6 shows us the performance of our models on generic datasets with the modern training recipe defined in Section 4.4 applied during training to obtain the source-trained model. We observe a similar pattern, as seen without these augmentations, repeated here. Self-attention transformer-based models outperform traditional convolutional architectures ResNet and HRNet, but ConvNeXt achieves the best or close to the best performance for both tasks on both datasets.

However, the image augmentations defined in our modern training recipe provide little to no improvement in terms of classification accuracy. In fact, at times, they even hurt the overall accuracy of the adaptation task. This can be attributed to the distortion introduced in the source images due to the strong augmentations. For standard datasets, such distortion leads to ambiguity in the otherwise unambiguous source data in terms of the centered positioning of the object, singular object in the frame, and non-interfering background, among others.

### 5.2. Aerial Datasets

The aerial datasets we evaluate consist of the DOTA-xView and the AID-UCM datasets, the formation of which is defined in Section 4.3. Table 7 shows the performance of our model backbones without the modern training recipe. Based on the results for the aerial datasets, we see a trend similar to standard datasets. Self-attention-based models tend to outperform convolutional models for the adaptation task. However, while ConvNeXt displays the best or close to the best performance for the generalization task, the SWIN backbone consistently outperforms all other models for the adaptation task.

Table 8 displays the results of our backbones on the aerial datasets with augmentations. In contrast to standard datasets, the modern training recipe image augmentations result in slightly increased performance for both generalization and adaptation tasks on aerial datasets. This can be attributed to larger variations in the appearance of the aerial images due to object positioning in the images, diverse angular rotations for each class, multiple objects in a frame, varying image contrast, etc. This is reflected in the increasing accuracy in Table 8.

### 5.3. Qualitative Analysis

**Figure 6:**
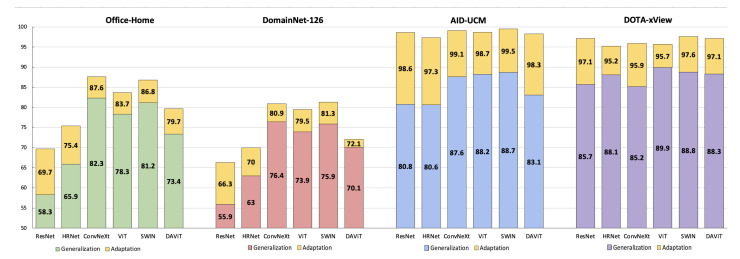
A bar chart overview of all our results across datasets after applying our modern training recipe, showing the performance of each backbone and the improvements gained by adaptation over generalization.

**Figure 7:**
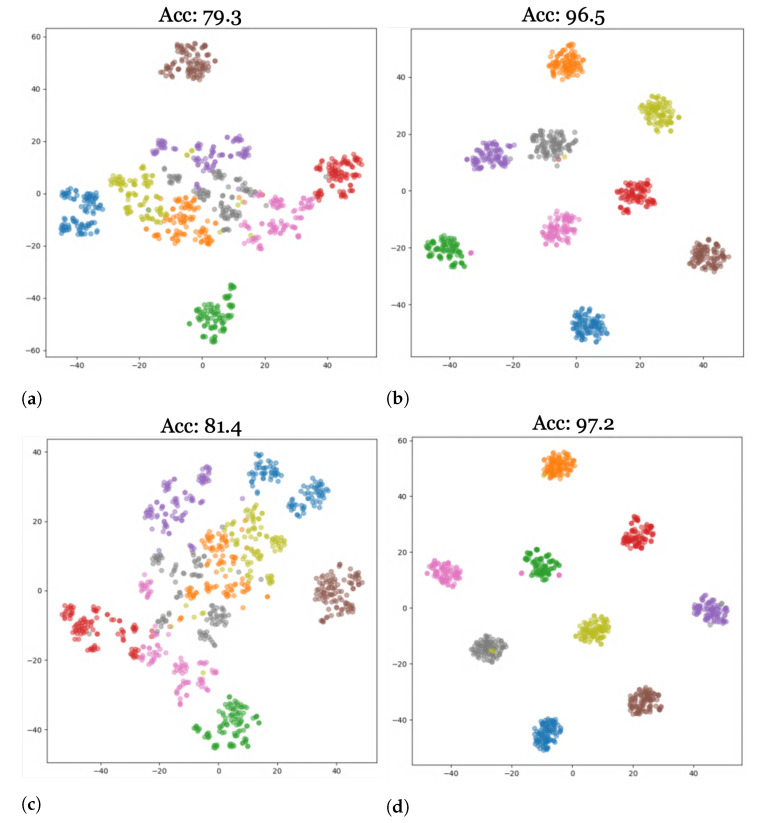
t-SNE plots for convolutional networks ResNet and HRNet on the AID-UCM dataset. (**a**) ResNet Generalization, (**b**) ResNet Adaptation, (**c**) HRNet Generalization, (**d**) HRNet Adaptation.

**Figure 8:**
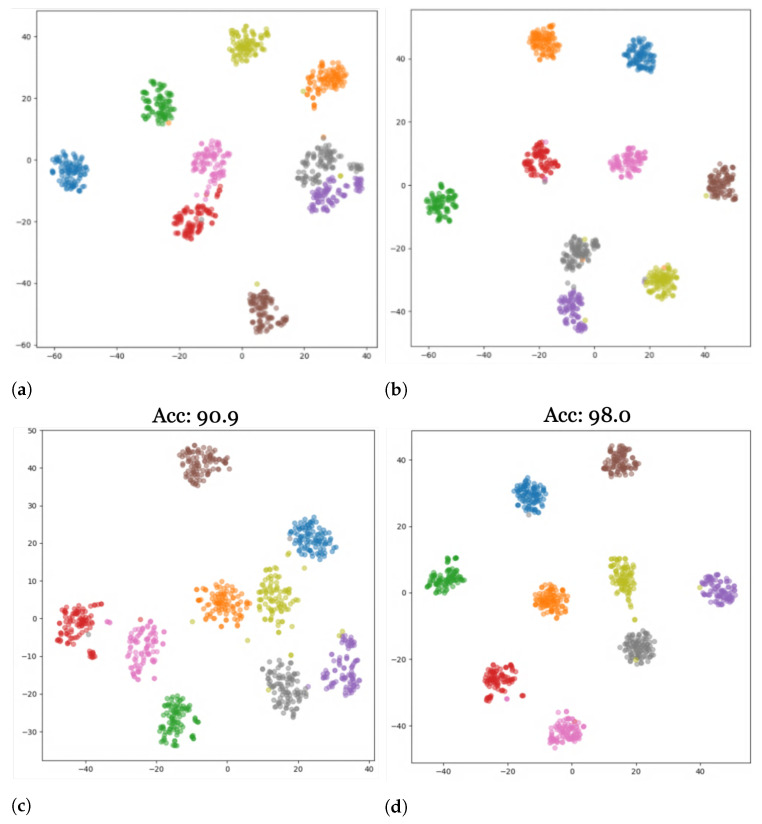
t-SNE plots for self-attention networks ConvNext and SWIN on the AID-UCM dataset. (**a**) ConvNeXt Generalization, (**b**) ConvNeXt Adaptation, (**c**) SWIN Generalization, (**d**) SWIN Adaptation.

In this section, we provide a qualitative discussion of our results. Figure 6 shows a graphical representation of our results for both standard and aerial datasets after applying our modernized training recipe. We additionally provide visualizations of the results of our study using t-distributed stochastic neighbor embeddings (t-SNE) [51]. t-SNE visualizes high-dimensional data in a 2-dimensional plot in three steps: first, it calculates the similarity between points in the higher-dimensional space, followed by a calculation of the distribution that measures the pairwise distances between the points in the lower-dimensional embedding space. Finally, KL-divergence is used to minimize the difference between the probability distributions of the higher- and lower-dimensional embedding spaces. Figure 7 and Figure 8 show plots for the source AID and target UCM datasets with the nine clusters corresponding to the nine classes defined in Table 3. The corresponding domain-wise results can be found in Appendix A.

Figure 7a,c visualize the features produced by the ResNet and HRNet, respectively, for the generalization task on the target data with the models trained on the source data only. Figure 7b,d provide the corresponding visualized features after adaptation. A significant tightening of clusters can be seen in the visualizations after adaptation, which is also reflected in the increased accuracies (~17% for ResNet and ~16% for HRNet).

Similarly, Figure 8 shows the results for self-attention-based models ConvNeXt (Figure 8a,b) and SWIN (Figure 8c,d) before and after adaptation from AID to UCM. Due to the higher generalization scores before adaptation, the increase in the cluster compactness and the inter-cluster distance in the feature space after adaptation are less prominent, but they are noticeably visible nonetheless.

It is understood that for a model to generalize to previously unseen domains, its predictions should not depend on features that are specific to the distribution of the training data since domain-specific features lead to a loss in accuracy when the model is provided with out-of-distribution data. Spurious features are often described in the literature [52] as any feature that correlates strongly with the labels in the training set, such as specific textures or colors in the background. Instead, the model should be able to utilize robust features that are invariant to covariate shift [53] and generalize well across other domains. Vision transformer architectures work on the conjecture that attention-based architectures are more likely to learn robust features from data compared to CNNs, given the ability of their self-attention blocks to communicate globally within a given input [1]. However, our results show that while a ConvNeXt backbone fares better in a sparse data setting (like Office-Home), the SWIN architecture shows a comparable or even better performance when provided with enough data. Moreover, our experiments with the modern training recipe verify that the augmentations help generalize better. This is especially apparent with aerial datasets. While self-attention based models require ample data to develop robust feature representations, with large enough datasets, they are able to generalize sufficiently well and adapt to other domains without a significant performance drop.

## 6. Conclusions

In this work, we provide an in-depth performance analysis of the effect of backbone architectures on domain generalization and unsupervised domain adaptation tasks for both standard and aerial datasets. Our experiments make use of the SHOT framework in order to perform unsupervised domain adaptation from the source domain to the target domain. We evaluate and compare the performance of convolutional and self-attention-based models, and observe that the architecture of the backbone plays a major role in generalization and adaptation to unseen domains. As shown in Section 5, self-attention based models like ViT, SWIN, and DAViT consistently outperform convolutional models ResNet and HRNet. However, the newer ConvNext model, which combines elements of convolutional and self-attention architectures, performs as well or better than transformer architectures and significantly better than standard CNNs on domain generalization and adaptation.

Given that model architectures like ViT are bottlenecked by the lack of large-scale datasets, we applied a set of well-crafted data augmentation techniques, which we termed a modern training recipe, to the source datasets to determine if such augmentations could optimize the performance of vision transformer models on cross-domain generalization. While these augmentations can adversely impact the performance of generic standard datasets, they can be helpful for aerial datasets.

## Figures and Tables

**Table 1:** Information about the backbone architectures used.

Model	Image Size	# Params
ResNet [7]	224×224	23 M
HRNet [8]	224×224	64 M
ConvNeXt [9]	224×224	89 M
ViT [1]	224×224	86 M
SWIN [2]	224×224	88 M
DAViT [3]	224×224	88 M

**Table 2:** The DOTA-xView domain adaptation dataset.

DOTA Classes	Number of Samples	Augmented Samples	xView Classes	Number of Samples	Augmented Samples
Plane	5000	0	Plane	1159	3841
Ship	5000	0	Ship	4476	524
Vehicle	5000	0	Vehicle	5000	0
Storage Tank	2126	2874	Storage Tank	1447	3553

**Table 3:** The AID-UCM domain adaptation dataset.

AID Classes	Number of Samples	UCM Classes	Number of Samples
Airport	360	Airplane	100
Parking	390	Parking Lot	100
Storage Tank	360	Storage Tank	100
Beach	400	Beach	100
Forest	350	Forest	100
River	410	River	100
Baseball Field	220	Baseball Diamond	100
Medium Residential	290	Medium Residential	100
Sparse Residential	300	Sparse Residential	100

**Table 4:** Generalization mean percent accuracy on Office-Home [13] with different combinations of four augmentations: RA (RandAugment), RE (random erasing), MX (Mixup), and CM (Cutmix). Bold shows best performance.

Backbone	RA + RE	RA + RE + MX	RA + RE + MX + CM
ResNet-50 [7]	**58.3**	55.9	55.9
HRNet48 [8]	**65.9**	63.6	61.3
ConvNeXt [9]	**82.3**	81.4	81.2
SWIN [2]	**81.2**	80.4	80.3

**Table 5:** Generalization and adaptation mean percent accuracy without augmentations on standard datasets.

Backbone	Generalization		Adaptation	
	Office-Home	DomainNet	Office-Home	DomainNet
ResNet [7]	59.9	56.4	69.2	67.4
HRNet [8]	66.1	62.5	75.9	70.2
ConvNeXt [9]	81.9	77.0	87.6	81.6
ViT [1]	73.9	72.2	83.4	77.9
SWIN [2]	81.6	76.7	87.0	81.7
DAViT [3]	73.4	70.0	79.8	73.2

**Table 6:** Generalization and adaptation mean percent accuracy with augmentations on standard datasets.

Backbone	Generalization		Adaptation	
	Office-Home	DomainNet	Office-Home	DomainNet
ResNet [7]	58.3	55.9	69.7	66.3
HRNet [8]	65.9	63.0	75.4	70.0
ConvNeXt [9]	82.3	76.4	87.6	80.9
ViT [1]	78.3	73.9	83.7	79.5
SWIN [2]	81.2	75.9	86.8	81.3
DAViT [3]	73.4	70.1	79.7	72.1

**Table 7:** Generalization and adaptation mean percent accuracy without augmentations on aerial datasets.

Backbone	Generalization		Adaptation	
	DOTA-xView	AID-UCM	DOTA-xView	AID-UCM
ResNet [7]	83.7	79.3	95.0	97.0
HRNet [8]	85.3	77.5	95.1	97.3
ConvNeXt [9]	89.1	88.2	96.1	98.2
ViT [1]	88.4	88.0	91.3	98.4
SWIN [2]	89.3	88.1	97.5	98.7
DAViT [3]	87.9	81.2	97.2	95.7

**Table 8:** Generalization and adaptation mean percent accuracy with augmentations on aerial datasets.

Backbone	Generalization		Adaptation	
	DOTA-xView	AID-UCM	DOTA-xView	AID-UCM
ResNet [7]	85.7	80.8	97.1	98.6
HRNet [8]	88.1	80.6	95.2	97.3
ConvNeXt [9]	85.2	87.6	95.9	99.1
ViT [1]	89.9	88.2	95.7	98.7
SWIN [2]	88.8	88.7	97.6	99.5
DAViT [3]	88.3	83.1	97.1	98.3

## Data Availability

Not applicable.

## Abbreviations

The following abbreviations are used in this manuscript:

CNN	Convolutional Neural Network
DA	Domain Adaptation
UDA	Unsupervised Domain Adaptation
SHOT	Source Hypothesis Transfer
ResNet	Residual Network
HRNet	High Resolution Network
ConvNext	Convolutional Network for the 2020s
ViT	Vision Transformer
SWIN	ShiftedWindow Vision Transformer
DAViT	Dual Attention Vision Transformer
DeiT	Data Efficient Image Transformer
DOTA	Dataset for Object Detection in Aerial Images
AID	Aerial Image Dataset
UCM	UC Merced Land Dataset
t-SNE	t-Distributed Stochastic Neighbor Embeddings

## Appendix A

This appendix contains detailed results for the experiments presented in Section 5.

### Appendix A.1. Office-Home

Table A1 and Table A2 present the performance comparison for all models on the Office-Home dataset without augmentations. We observe that self-attention based models, such as ViT and SWIN, generally outperformed traditional convolutional models in both tasks. However, the ConvNeXt backbone, which is a modified convolutional architecture, achieved higher average accuracy compared to the best-performing vision transformer.

**Table A1:** Mean percent accuracy for generalization without augmentations on Office-Home [13] (source → target).

Backbone	Ar → Cl	Ar → Pr	Ar → Re	Cl → Ar	Cl → Pr	Cl → Re	Pr → Ar	Pr → Cl	Pr → Re	Re → Ar	Re → Cl	Re → Pr	Avg.
ResNet-50 [7]	44.5	65.6	74.1	54.0	61.2	64.7	52.1	41.2	73.3	65.1	46.2	78.0	60.0
HRNet48 [8]	50.5	71.6	79.5	62.0	70.2	71.1	61.4	46.3	78.8	70.5	52.2	80.0	66.2
ConvNeXt [9]	72.9	86.0	89.3	82.6	85.2	87.5	80.2	67.9	89.9	82.6	70.2	89.7	82.0
ViT [1]	50.5	82.5	86.8	74.3	82.7	84.3	73.2	50.4	88.2	77.7	49.8	87.6	74.0
SWIN [2]	70.1	85.2	89.0	82.3	86.8	88.1	80.0	66.4	89.4	83.3	68.4	90.4	81.6
DAViT [3]	58.0	79.1	84.4	73.4	79.5	81.2	70.6	54.4	83.2	75.8	56.0	85.9	73.4

**Table A2:** Mean percent accuracy for adaptation without augmentations on Office-Home [13] (source → target).

Backbone	Ar → Cl	Ar → Pr	Ar → Re	Cl → Ar	Cl → Pr	Cl → Re	Pr → Ar	Pr → Cl	Pr → Re	Re → Ar	Re → Cl	Re → Pr	Avg.
ResNet-50 [7]	48.9	72.0	75.5	64.8	76.5	75.2	67.7	53.9	82.3	72.2	58.6	82.7	69.2
HRNet48 [8]	61.4	80.11	84.7	74.4	80.8	82.5	75.7	60.3	84.7	77.8	63.4	85.9	76.0
ConvNeXt [9]	79.2	92.4	92.2	88.4	92.1	92.3	86.6	77.6	92.3	87.6	78.7	92.8	87.7
ViT [1]	69.8	89.6	90.0	84.8	89.6	89.5	83.8	67.5	90.5	84.5	69.7	92.3	83.4
SWIN [2]	76.2	91.5	91.7	87.4	92.0	92.0	86.6	77.0	91.7	87.9	76.6	94.2	87.1
DAViT [3]	65.3	85.7	86.9	81.0	87.1	86.4	79.6	63.8	86.5	80.5	66.8	88.6	79.8

Furthermore, as seen in Table A3 and Table A4, we notice a similar trend in performance even after we apply the augmentations defined in Section 4.4. We see that ConvNeXt outperforms all other models, both convolutional and attention based.

**Table A3:** Mean percent accuracy for generalization with augmentations on Office-Home [13] (source → target).

Backbone	Ar → Cl	Ar → Pr	Ar → Re	Cl → Ar	Cl → Pr	Cl → Re	Pr → Ar	Pr → Cl	Pr → Re	Re → Ar	Re → Cl	Re → Pr	Avg.
ResNet-50 [7]	45.0	63.1	71.3	47.2	58.3	59.5	51.9	43.0	72.1	63.8	47.5	76.3	58.3
HRNet48 [8]	52.3	70.7	78.0	58.5	68.3	69.8	61.6	49.0	78.0	70.3	55.1	79.7	65.9
ConvNeXt [9]	70.8	86.6	89.0	83.7	86.0	88.2	80.2	69.2	89.0	83.3	71.5	90.2	82.3
ViT [1]	63.7	83.2	87.3	79.1	83.5	86.6	75.6	59.8	87.2	80.6	64.4	88.8	78.3
SWIN [2]	69.5	85.5	88.9	81.2	85.5	87.4	79.2	66.1	88.9	82.8	69.1	90.3	81.2
DAViT [3]	57.3	79.3	84.3	72.7	79.1	80.7	70.7	54.0	83.5	76.8	56.7	85.8	73.4

**Table A4:** Mean percent accuracy for adaptation with augmentations on Office-Home [13] (source → target).

Backbone	Ar → Cl	Ar → Pr	Ar → Re	Cl → Ar	Cl → Pr	Cl → Re	Pr → Ar	Pr → Cl	Pr → Re	Re → Ar	Re → Cl	Re → Pr	Avg.
ResNet-50 [7]	55.9	74.4	79.2	64.9	74.7	73.9	65.6	54.6	80.7	71.6	58.6	82.7	69.7
HRNet48 [8]	60.7	80.0	83.9	73.2	79.2	80.4	75.9	62.0	84.6	76.8	63.2	84.7	75.4
ConvNeXt [9]	77.5	92.0	91.8	88.5	91.9	91.7	87.6	77.4	92.4	87.8	79.4	93.9	87.6
ViT [1]	71.9	88.8	89.8	84.5	90.0	89.9	82.4	69.4	90.2	84.6	70.5	92.1	83.7
SWIN [2]	77.4	91.1	91.7	87.2	91.6	91.9	86.9	75.6	91.7	86.9	76.8	93.6	86.8
DAViT [3]	64.7	86.7	86.7	79.4	86.5	86.4	78.9	64.7	86.7	81.7	65.6	88.3	79.7

While these results hold true for all practical purposes, the Office-Home dataset provides a limited amount of data for model training. Averaging around 70 images per class, and with a maximum of 99 images in a class in the Clipart domain, transformer models like SWIN may not have enough data to develop appropriate and adaptable feature representations. Thus, we turn to the larger DomainNet dataset to better understand the dependency of models on data for unsupervised domain adaptation tasks.

### Appendix A.2. DomainNet

The DomainNet dataset, with nearly 0.5 million images, offers a much denser and more diverse dataset for our models to train on. With more data available during training, we notice a subtle difference in the trend compared to the Office-Home results.

Table A5 and Table A6 present the performance for all models on the larger DomainNet dataset without augmentations. We conjecture that with an increase in data, self-attention-based models, such as SWIN, are able to measure up to the performance of the ConvNeXt backbone for adaptation. Our findings are confirmed in Table A7 and Table A8.

**Table A5:** Mean percent accuracy for generalization without augmentations on DomainNet [14] (source → target).

Backbone	C → P	C → R	C → S	P → C	P → R	P → S	R → C	R → P	R → S	S → C	S → P	S → R	Avg.
ResNet-50 [7]	47.7	61.3	48.7	55.0	74.8	50.0	57.6	63.4	48.6	57.1	52.5	60.2	56.4
HRNet48 [8]	55.6	68.0	56.9	62.7	79.1	55.9	61.9	66.9	53.0	64.1	59.4	66.9	62.5
ConvNeXt [9]	74.0	83.3	72.6	75.8	88.2	70.8	74.8	79.2	68.5	76.8	76.4	83.5	77.0
ViT [1]	70.7	80.7	67.4	70.7	85.9	60.3	67.7	74.5	58.2	74.7	74.2	82.0	72.2
SWIN [2]	74.2	83.7	71.5	76.3	88.0	68.5	75.1	78.5	67.6	77.4	75.9	83.8	76.7
DAViT [3]	66.4	76.3	65.3	68.0	83.4	63.6	67.0	73.7	60.6	70.8	68.7	76.3	70.0

**Table A6:** Mean percent accuracy for adaptation without augmentations on DomainNet [14] (source → target).

Backbone	C → P	C → R	C → S	P → C	P → R	P → S	R → C	R → P	R → S	S → C	S → P	S → R	Avg.
ResNet-50 [7]	61.2	77.3	59.7	68.4	80.1	61.1	68.1	66.3	58.2	68.8	63.2	76.4	67.4
HRNet48 [8]	66.0	79.5	64.1	70.8	82.1	64.5	69.6	69.4	61.5	71.6	65.8	77.5	70.3
ConvNeXt [9]	79.2	90.1	76.5	79.4	90.4	77.1	79.5	81.4	76.1	79.5	80.5	89.5	81.6
ViT [1]	76.2	86.9	71.8	77.5	87.9	70.9	74.7	77.1	66.5	80.2	78.2	87.7	77.9
SWIN [2]	79.8	90.0	75.0	80.9	90.2	76.2	81.2	81.1	74.5	82.0	80.1	89.8	81.7
DAViT [3]	70.2	82.9	68.0	71.1	83.1	66.5	71.9	73.6	66.7	71.8	71.0	82.1	73.2

**Table A7:** Mean percent accuracy for generalization with augmentations on DomainNet [14] (source → target).

Backbone	C → P	C → R	C → S	P → C	P → R	P → S	R → C	R → P	R → S	S → C	S → P	S → R	Avg.
ResNet-50 [7]	45.3	59.5	49.7	55.2	73.9	51.5	55.0	63.4	49.9	55.9	52.2	59.3	55.9
HRNet48 [8]	55.5	66.9	57.9	63.1	78.4	57.9	61.9	68.4	56.1	64.8	59.3	66.5	63.0
ConvNeXt [9]	72.4	83.0	70.2	75.6	88.1	69.1	74.5	79.4	69.1	76.6	76.0	83.2	76.4
ViT [1]	69.9	80.4	68.8	73.4	87.0	64.4	71.8	76.7	62.3	76.5	74.2	81.9	73.9
SWIN [2]	72.6	83.3	69.0	75.6	87.9	68.2	74.5	78.1	68.7	76.5	74.8	83.3	75.9
DAViT [3]	65.6	79.9	63.8	67.9	80.3	64.5	69.3	68.8	62.7	68.2	69.9	0.1	70.1

**Table A8:** Mean percent accuracy for adaptation with augmentations on DomainNet [14] (source → target).

Backbone	C → P	C → R	C → S	P → C	P → R	P → S	R → C	R → P	R → S	S → C	S → P	S → R	Avg.
ResNet-50 [7]	59.8	75.6	58.3	68.2	78.8	60.4	67.7	64.9	59.1	67.3	61.2	74.0	66.3
HRNet48 [8]	65.3	78.9	64.7	70.4	81.7	65.2	69.6	69.8	62.8	71.1	64.5	75.9	70.0
ConvNeXt [9]	78.9	90.0	76.0	78.6	90.3	76.9	79.3	80.3	75.1	78.9	80.1	86.9	80.9
ViT [1]	77.4	87.9	73.0	78.9	89.0	72.3	78.5	79.7	69.6	80.9	78.7	88.0	79.5
SWIN [2]	79.1	90.0	74.8	80.6	90.3	75.5	80.9	79.9	74.4	80.4	79.0	90.2	81.3
DAViT [3]	70.2	82.9	68.0	71.1	83.1	66.5	71.9	73.6	66.7	71.8	71.0	82.1	73.2

Table A7 and Table A8 report the performance of our models for generalization and adaptation on the DomainNet dataset with augmentations. Our modern training recipe augmentations, described in Section 4.4), further diversify DomainNet via RandAugment and RandErase. While SWIN backbone displays comparable performance to ConvNeXt without augmentations, source training SWIN with even more diversified data marginally outperforms the ConvNeXt model for the adaptation task. This further solidifies our hypothesis that self-attention-based models are able to develop more robust feature representations compared to convolutional backbones, provided they are given enough data.

### Appendix A.3. DOTA-xView

We now examine how our models perform on aerial datasets. Table A9 and Table A10 show the results of generalization and adaptation on the DOTA-xView dataset described in Section 4.3.1.

**Table A9:** Mean percent accuracy for generalization without augmentations on DOTA-xView (source → target).

Backbone	DOTA → xView	xView → DOTA	Avg.
ResNet [7]	79.1	88.4	83.7
HRNet [8]	81.5	89.0	85.3
ConvNeXt [9]	84.1	94.0	89.1
ViT [1]	81.6	95.3	88.4
SWIN [2]	86.6	91.9	89.3
DAViT [3]	86.6	89.1	87.9

**Table A10:** Mean percent accuracy for adaptation without augmentations on DOTA-xView (source → target).

Backbone	DOTA → xView	xView → DOTA	Avg.
ResNet [7]	90.7	99.3	95.0
HRNet [8]	91.0	99.1	95.1
ConvNeXt [9]	92.8	99.4	96.1
ViT [1]	84.7	97.9	91.3
SWIN [2]	95.4	99.5	97.5
DAViT [3]	94.9	99.5	97.2

Similar to the results on DomainNet, we notice that our attention-based backbones outperform convolutional models for both tasks. Given the abundance of data in our shared dataset (as seen in Table 2), this further solidifies our previous inference that the amount of data used for training and adaptation is directly related to the classification accuracy.

Table A11 and Table A12 present the performance of our models on the DOTA-xView dataset with augmentations for the generalization and adaptation tasks. Again, we observe a similar trend of attention-based models outperforming convolutional models.

**Table A11:** Mean percent accuracy for generalization with augmentations on DOTA-xView (source → target).

Backbone	DOTA → xView	xView → DOTA	Avg.
ResNet [7]	78.3	91.9	85.1
HRNet [8]	83.2	92.9	88.1
ConvNeXt [9]	82.3	88.1	85.2
ViT [1]	89.0	90.9	89.9
SWIN [2]	87.7	89.9	88.8
DAViT [3]	85.2	91.3	88.3

**Table A12:** Mean percent accuracy for adaptation with augmentations on DOTA-xView (source → target).

Backbone	DOTA → xView	xView → DOTA	Avg.
ResNet [7]	94.5	99.6	97.1
HRNet [8]	92.0	98.4	95.2
ConvNeXt [9]	92.4	99.5	95.9
ViT [1]	94.2	97.2	95.7
SWIN [2]	95.4	99.7	97.6
DAViT [3]	94.6	99.7	97.1

### Appendix A.4. AID-UCM

The final dataset on which we show results is the AID-UCM dataset, the specifics of which are defined in Section 4.3.2. Table A13 and Table A14 present the results for generalization and adaptation for all models.

**Table A13:** Mean percent accuracy for generalization without augmentations on AID-UCM (source → target).

Backbone	AID → UCM	UCM → AID	Avg.
ResNet [7]	81.2	77.5	79.3
HRNet [8]	81.4	73.7	77.5
ConvNeXt [9]	91.6	84.8	88.2
ViT [1]	94.3	81.7	88.0
SWIN [2]	90.9	84.1	87.5
DAViT [3]	84.8	77.7	81.2

**Table A14:** Mean percent accuracy for adaptation without augmentations on AID-UCM (source → target).

Backbone	AID → UCM	UCM → AID	Avg.
ResNet [7]	96.5	97.5	97.0
HRNet [8]	97.2	97.5	97.3
ConvNeXt [9]	98.3	98.1	98.2
ViT [1]	98.1	98.7	98.4
SWIN [2]	98.0	98.7	98.7
DAViT [3]	98.2	93.3	95.7

Table A15 and Table A16 present our results on the AID-UCM dataset after applying our model training recipe. We can see that attention-based models consistently display a higher classification accuracy as compared to the convolutional models.

**Table A15:** Mean percent accuracy for generalization with augmentations on AID-UCM (source → target).

Backbone	AID → UCM	UCM → AID	Avg.
ResNet [7]	87.6	74.0	80.8
HRNet [8]	90.2	71.1	80.7
ConvNeXt [9]	92.1	83.1	87.6
ViT [1]	94.2	82.2	88.2
SWIN [2]	94.9	82.5	88.7
DAViT [3]	90.5	75.8	83.1

**Table A16:** Mean percent accuracy for adaptation with augmentations on AID-UCM (source → target).

Backbone	AID → UCM	UCM → AID	Avg.
ResNet [7]	99.1	98.2	98.6
HRNet [8]	98.1	96.6	97.3
ConvNeXt [9]	99.2	99.0	99.1
ViT [1]	98.4	99.0	98.7
SWIN [2]	99.6	99.4	99.5
DAViT [3]	99.1	97.6	98.3
